# Anxiety among hospitalized COVID-19 patients: a case–control study from a tertiary teaching hospital in Malaysia

**DOI:** 10.3389/fpsyt.2023.1148019

**Published:** 2023-05-18

**Authors:** Hui Jan Tan, Abdool Alleem Hj Shahren, Ching Soong Khoo, Chen Fei Ng, Wan Asyraf Wan Zaidi, Najma Kori, Petrick Periyasamy, Choon Leng Eu, Alvin Oliver Payus, Rozita Hod

**Affiliations:** ^1^Department of Medicine, Faculty of Medicine, The National University of Malaysia, Kuala Lumpur, Malaysia; ^2^Department of Psychiatry, Faculty of Medicine, The National University of Malaysia, Kuala Lumpur, Malaysia; ^3^Department of Medicine, Faculty of Medicine, University Malaysia Sabah, Kota Kinabalu, Malaysia; ^4^Department of Community Health, Faculty of Medicine, The National University of Malaysia, Kuala Lumpur, Malaysia

**Keywords:** anxiety, COVID-19, hospitalized, case control, Generalized Anxiety Disorder-7

## Abstract

**Introduction:**

Anxiety has been increasingly recognized as part of the psychosocial health issues in COVID-19 patients. However, the impact of this topic may be underestimated in low- and middle-income countries. This study aimed to estimate the prevalence of and risk factors of anxiety in COVID-19 patients compared to controls in a local tertiary teaching hospital in Malaysia.

**Methods:**

In this case–control study, we analyzed data on adult patients aged 18 years and above hospitalized for COVID-19 infection with matched hospitalized controls. The demographic, clinical data and anxiety measures using the Generalized Anxiety Disorder-7 questionnaire were analyzed using univariate and multivariate analysis.

**Results:**

86.6% in the COVID-19 group had anxiety, significantly higher than 13.4% in the control group (*p* = 0.001). The COVID-19 group was significantly associated with the GAD-7 severity (*p* = 0.001). The number of COVID-19 patients in the mild, moderate, and severe anxiety groups was 48 (84.2%), 37 (86%), and 18 (94.7%), respectively. Multiple logistic regression showed significant predictors for anxiety, including COVID-19 diagnosis and neurological symptoms. Anxiety was found 36.92 times higher in the patients with COVID-19 compared to those without COVID-19 (OR 36.92;95% CI 17.09, 79.78, *p* = 0.001). Patients with neurological symptoms were at risk of having anxiety (OR 2.94; 95% CI 1.03, 8.41, *p* = 0.044).

**Discussion:**

COVID-19 patients experience a significant disruption in psychosocial functioning due to hospitalization. The burden of anxiety is notably high, compounded by a diagnosis of COVID-19 itself and neurological symptomatology. Early psychiatric referrals are warranted for patients at risk of developing anxiety symptoms.

## 1. Introduction

There has been a growing recognition of neuropsychiatric manifestations since the declaration of the COVID-19 pandemic. The two most prevalent disabling mental disorders were depressive and anxiety disorders. A meta-analysis of mental health burden following the impact of COVID-19 showed that the prevalence of anxiety was 27.77% (CI: 24.47–31.32) (1). Previous studies have reported prevalence rates of anxiety between 18.6% (2) –34.72% (3). Hospitalized COVID-19 patients invariably have high anxiety levels from multifactorial etiology. This has been reflected following high anxiety levels in hospitalized COVID-19 patients in Turkey (4) and Iran (5). Anxiety is associated with specific stressors in hospitalized patients, which the additional burden of COVID-19 may further compound. Factors such as uncertainty, the inadequacy of explanation, isolation from family, physical effects of the illness, and financial worries may cause formidable barriers in this vulnerable group.

The South East Asia region had recorded 57 million confirmed cases and more than half a million deaths from COVID-19 infections (6). Malaysia had reported approximately 30,000 deaths by December 2021 (7). The impact of COVID-19 has not only resulted in a financial burden but also caused a sharp rise in psychological disorders in the population. Previous studies in Malaysia have focused on specific groups: healthcare workers (8, 9), children with autism (10), women (11), general population (12), urban and rural communities (13) and university students (14). There is a paucity of literature that compared anxiety levels between COVID-19 patients and other hospitalized medical patients, especially from this region.

Determining variables associated with anxiety in COVID-19 patients may be impeded by several issues, such as coexisting medical illnesses, clinical features, the severity and complications of the disease, and the duration of hospitalization or quarantine period. However, it is crucial to determine factors associated with developing anxiety so that we can address them earlier. This knowledge gap remains problematic as it is more challenging to perform studies in hospitalized COVID-19 patients who may also suffer from complications of the disease and its psychological effects. To address this gap, we embark on this study to determine the prevalence and risk factors of anxiety in COVID-19 patients compared to non-COVID-19 patients as controls in a tertiary teaching hospital in Malaysia.

## 2. Materials and methods

### 2.1. Study design and study population

This case–control study was conducted between 1 June 2021 and 31 December 2022 at Hospital Canselor Tuanku Muhriz, National University of Malaysia. This tertiary teaching hospital has received COVID-19 cases since the pandemic began in 2020.

The study population was recruited *via* simple random sampling and patients had to fulfill the following criteria: (1) Patients more than 18 years old with a diagnosis of COVID-19 *via* qualitative reverse transcription polymerase chain reaction (RT-PCR) from nasopharyngeal and/or oropharyngeal swab, and (2) Hospitalized patients. The controls were matched to the cases by gender and age. They were hospitalized patients in the medical wards due to other medical conditions apart from COVID-19 infection.

### 2.2. Data collection

Upon admission, consent was taken from the patients or the next of kin/caregivers. The patients/caregivers were given a set of questions to be answered on a virtual questionnaire according to their suited language (English and Bahasa Malaysia). The clinical and laboratory investigation data were further collated.

### 2.3. Ethical statement

This study was conducted following the guidelines in the Declaration of Helsinki and was approved by the Ethics and Research Board of the Faculty of Medicine, the National University of Malaysia FF-2021-379.

### 2.4. Questionnaire sections

#### 2.4.1. Demographic variables

This section explored demographic and occupational characteristics. The demographic variables included age, gender, marital status, occupation, habits (smoking and alcohol), and education level.

#### 2.4.2. Clinical variables

The second section explored the clinical characteristics of comorbidities, presenting symptoms, and laboratory parameters. The presenting symptoms were divided into respiratory symptoms (fever, runny nose, sore throat, shortness of breath, and cough), gastrointestinal symptoms (diarrhea, vomiting, abdominal pain, and poor oral intake), neurological symptoms (seizures, limb weakness, headache, and dizziness), and musculoskeletal symptoms (muscle and joint pains). The severity of patients with COVID-19 was divided to five clinical categories according to our local guidelines (15): 1—asymptomatic, 2—symptomatic, 3—evidence of pneumonia, 4—oxygen supplement requirement, and 5—intubated and/or multiorgan failure. In addition, the laboratory data were retrieved from the hospital’s data management system.

#### 2.4.3. Anxiety variable

The third section consists of the study instrument which was a questionnaire on General Anxiety Disorder-7 (GAD-7) in English (16) and the validated Bahasa Malaysia version (17). The patients had the alternative to answer in English or the Bahasa Malaysia version. The GAD-7 questionnaire is a 7-item, self-reporting anxiety questionnaire designed to evaluate mental health symptoms. The questionnaire inquires about the degree to which the patient has been bothered by feeling nervous, anxious, on edge; not being able to stop or control worrying; worrying too much about different things; having trouble relaxing; being so restless that it is hard to sit still; becoming easily annoyed or irritable; and feeling afraid as if something awful might happen. This scale consists of 7 questions responded on a four-point Likert scale ranging from 0 (not at all), 1 (several days), 2 (more than half the days) to 3 (nearly every day). GAD-7 total score for the seven items ranged from 0 to 21. A total score of 0–4 indicates minimal anxiety, 5–9 indicates mild anxiety, 10–14 indicates moderate anxiety, and 15–21 indicates severe anxiety. The GAD-7 is a valid and efficient tool for screening for GAD and assessing its severity in clinical practice and research with 89% sensitivity and 82% specificity. Previous studies that employed similar tools showed a prevalence rate of 17.9–22.6% for GAD during the COVID-19 outbreak (18, 19).

### 2.5. Statistical analysis

Data were analyzed using the software SPSS Statistic for Windows, version 25. Data normality was evaluated using one-sample Kolmogorov–Smirnov presented as median ± interquartile range for skewed data and frequency (percentage) for nominal data. The demographic factors and clinical characteristics (categorical variables) were analyzed using the Chi-square test. The variables were divided into demographic, clinical (clinical characteristics and laboratory investigations), and anxiety variables. The multivariate logistic regression analysis was performed by including the variables with a value of p less than 0.05 from the simple logistic regression analysis.

## 3. Results

### 3.1. Demographic variables

The distribution of the demographic characteristics is shown in Table 1. Of the 223 patients, 118 were COVID-19 positive, and 105 were in the control group. Overall, the median (IQR) age of the COVID-19 and control groups was 54 (40.75, 65) years and 56 (37.50, 68) years, respectively (*p* = 0.975; Table 2). There was no significant difference in patients’ age, gender, marital status, race, habits, and education level. Only the employment status was significantly different between both groups, where the number of those who were employed in the COVID-19 group was higher (57, 67.1%) compared to the control group (28, 32.9%; *p* = 0.001). The median (IQR) length of hospitalization was significantly higher in the COVID-19 group, 12 (8.00, 20.25) compared to the control group, 10 (5.00, 17.50; *p* = 0.032; Table 2).

**Table 1:** Demographic, clinical characteristics, GAD-7 score, and laboratory investigations of the study population and controls.

		Total	Control *n* (%)	COVID-19 *n* (%)	*χ* ^2^	*p*
*Demographic variables*
Age group (years)	15–64	157	73 (46.5)	84 (53.5)	0.07	0.786	> 65	66	32 (48.5)	34 (51.5)		
Gender	Male	141	68 (48.2)	73 (51.8)	0.20	0.654	Female/	82	37 (45.1)	45 (54.9)		
Marital status	Single	41	21 (51.2)	20 (48.8)	0.35	0.557	Married	182	84 (46.2)	98 (53.8)		
Ethnic group	Malay	138	66 (47.8)	72 (52.2)	0.41	0.937	Chinese	52	24 (46.2)	28 (53.8)			Indian	18	9 (50)	9 (50)			Others	15	6 (40)	9 (60)		
Habits	None	182	82 (45.1)	100 (54.9)	5.90	0.117	Smoking	34	21 (61.8)	13 (38.2)			Alcohol	2	0 (0)	2 (100)			Smoking and Alcohol	5	2 (40)	3 (60)		
Education level	None	6	3 (50)	3 (50)	1.01	0.799	Primary	58	29 (50)	29 (50)			Secondary	133	63 (47.4)	70 (52.6)			University	26	10 (38.5)	16 (61.5)		
Employment	Unemployed	138	77 (55.8)	61 (44.2)	11.03	0.001*	Employed	85	28 (32.9)	57 (67.1)		
*Clinical characteristics*
Respiratory symptoms (fever, runny nose, sore throat, shortness of breath, and cough)	No	57	44 (77.2)	13 (22.8)	26.26	0.001*	Yes	166	61 (36.7)	105 (63.3)		
Fever	No	99	61 (61.6)	38 (38.4)	14.06	0.001*	Yes	124	44 (35.5)	80 (64.5)		
Runny nose	No	205	105 (51.2)	100 (48.8)	15.43	0.001*	Yes	18	0 (0)	18 (100)		
Sore throat	No	193	105 (54.4)	88 (45.6)	28.70	0.001*	Yes	30	0 (0)	30 (100)		
Shortness of breath	No	155	89 (57.4)	66 (42.6)	21.79	0.001*	Yes	68	16 (23.5)	52 (76.5)		
Cough	No	134	91 (67.9)	43 (32.1)	58.44	0.001*	Yes	89	14 (15.7)	75 (84.3)		
Gastrointestinal symptoms (diarrhea, vomiting, abdominal pain, and poor intake)	No	207	93 (44.9)	114 (55.1)	5.39	0.020*	Yes	16	12 (75)	4 (25)		
Diarrhea	No	218	103 (47.2)	115 (52.8)	0.00	1.000	Yes	5	2 (40)	3 (60)		
Vomiting	No	220	102 (46.4)	118 (53.6)	1.60	0.205	Yes	3	3 (100)	0 (0)		
Abdominal pain	No	216	99 (45.8)	117 (54.2)	2.88	0.090	Yes	7	6 (85.7)	1 (14.3)		
Poor intake	No	219	102 (46.6)	117 (53.4)	0.39	0.533	Yes	4	3 (75)	1 (25)		
Neurological symptoms (seizures, weakness, headache, and dizziness)	No	181	93 (51.4)	88 (48.6)	7.12	0.008*	Yes	42	12 (28.6)	30 (71.4)		
Seizures	No	220	103 (46.8)	117 (53.2)	0.01	0.919	Yes	3	2 (66.7)	1 (33.3)		
Weakness	No	217	100 (46.1)	117 (53.9)	1.93	0.165	Yes	6	5 (83.3)	1 (16.7)		
Headache	No	197	103 (52.3)	94 (47.7)	16.58	0.001*	Yes	26	2 (7.7)	24 (92.3)		
Dizziness	No	212	102 (48.1)	110 (51.9)	1.08	0.298	Yes	11	3 (27.3)	8 (72.7)		
Muscle and joint pain	No	206	104 (50.5)	102 (49.5)	10.81	0.001*	Yes	17	1 (5.9)	16 (94.1)		
*Comorbidities*
Diabetes mellitus	No	139	58 (41.7)	81 (58.3)	4.25	0.039*	Yes	84	47 (56)	37 (44)		
Hypertension	No	125	56 (44.8)	69 (55.2)	0.60	0.440	Yes	98	49 (50)	49 (50)		
Chronic kidney disease	No	193	87 (45.1)	106 (54.9)	2.32	0.128	Yes	30	18 (60)	12 (40)		
Dyslipidemia	No	189	91 (48.1)	98 (51.9)	0.56	0.453	Yes	34	14 (41.2)	20 (58.8)		
Ischemic heart disease	No	191	89 (46.6)	102 (53.4)	0.13	0.721	Yes	32	16 (50)	16 (50)		
Bronchial asthma	No	208	102 (49)	106 (51)	3.64	0.056	Yes	15	3 (20)	12 (80)		
*Anxiety variable*
GAD-7 score	minimal anxiety	104	89 (85.6)	15 (14.4)	115.90	0.001*	Anxiety	119	16 (13.4)	103 (86.6)		
GAD-7 severity	Minimal anxiety	104	89 (85.6)	15 (14.4)	130.26	0.001*	Mild anxiety	57	9 (15.8)	48 (84.2)			Moderate anxiety	43	6 (14)	37 (86)			Severe anxiety	19	1 (5.3)	18 (94.7)		
*Laboratory investigations*
Hemoglobin g/dL	12.0–15.0	99	39 (39.4)	60 (60.6)	4.23	0.040*	Abnormal	124	66 (53.2)	58 (46.8)		
White cell count x10^9^/L	4.0–10.0	124	48 (38.7)	76 (61.3)	7.86	0.005*	Abnormal	99	57 (57.6)	42 (42.4)		
Platelet x10^9^/L	150–410	167	76 (45.5)	91 (54.5)	0.66	0.415	Abnormal	56	29 (51.8)	27 (48.2)		
Sodium mmol/L	136–145	115	61 (53)	54 (47)	3.38	0.066	Abnormal	108	44 (40.7)	64 (59.3)		
Potassium mmol/L	3.5–5.1	176	88 (50)	88 (50)	2.85	0.092	Abnormal	47	17 (36.2)	30 (63.8)		
Urea mmol/L	2.5–6.7	129	57 (44.2)	72 (55.8)	1.03	0.310	Abnormal	94	48 (51.1)	46 (48.9)		
Creatinine μmol/L	50.4–98.1	124	53 (42.7)	71 (57.3)	2.12	0.146	Abnormal	99	52 (52.5)	47 (47.5)		
Total protein g/L	64–83	170	78 (45.9)	92 (54.1)	0.42	0.519	Abnormal	53	27 (50.9)	26 (49.1)		
Albumin g/L	34–48	106	49 (46.2)	57 (53.8)	0.06	0.807	Abnormal	117	56 (47.9)	61 (52.1)		
Bilirubin μmol/L	3.4–20.5	183	85 (46.4)	98 (53.6)	0.17	0.683	Abnormal	40	20 (50)	20 (50)		
Alanine transaminase IU/L	0–55	180	93 (51.7)	87 (48.3)	7.86	0.005^*^	Abnormal	43	12 (27.9)	31 (72.1)		
Alkaline phosphatase IU/L	40–150	191	89 (46.6)	102 (53.4)	0.13	0.721	Abnormal	32	16 (50)	16 (50)		

* Significant *p* < 0.05; *χ*^2^, Chi-square test; GAD-7, Generalized Anxiety Disorder-7.

**Table 2:** Comparison of the variables between the study population and controls.

			Percentiles					
	Group	*N*	50th (Median)	25th	75th	IQR	U	*p*
Age (years)	Control	105	56.00	37.50	68.00	30.50	6180.00	0.975	Covid 19	118	54.00	40.75	65.00	24.25		
GAD-7 Score	Control	105	1.00	0.00	2.00	2.00	1410.50	0.001^*^	Covid 19	118	8.00	7.00	14.00	7.00		
Length of hospitalization	Control	105	10.00	5.00	17.50	12.50	5163.50	0.032^*^	Covid 19	118	12.00	8.00	20.25	12.25		
Hemoglobin level (g/dL)	Control	105	12.10	10.15	14.05	3.90	5062.50	0.019^*^	Covid 19	118	13.40	11.48	14.73	3.25		
White cell count × 10^9^/L	Control	105	10.30	8.05	15.10	7.05	4108.50	0.001^*^	Covid 19	118	8.50	5.98	10.50	4.53		
Platelet × 10^9^/L	Control	105	276.00	208.00	345.00	137.00	4881.50	0.006^*^	Covid 19	118	236.50	177.75	289.00	111.25		
Sodium mmol/L	Control	105	136.00	133.00	139.00	6.00	5174.00	0.033^*^	Covid 19	118	135.00	131.00	138.00	7.00		
Potassium mmol/L	Control	105	4.00	3.70	4.40	0.70	5264.50	0.053	Covid 19	118	3.90	3.58	4.30	0.73		
Urea mmol/L	Control	105	5.50	3.80	10.20	6.40	5619.00	0.231	Covid 19	118	4.90	3.30	7.63	4.33		
Creatinine μmol/L	Control	105	97.20	75.65	180.40	104.75	5196.00	0.038^*^	Covid 19	118	86.80	72.75	129.83	57.08		
Total protein g/L	Control	105	71.00	65.50	78.00	12.50	6172.50	0.963	Covid 19	118	72.50	66.00	78.00	12.00		
Albumin g/L	Control	103	33.00	27.00	38.00	11.00	6031.00	0.923	Covid 19	118	33.00	29.00	37.00	8.00		
Bilirubin μmol/L	Control	105	12.30	8.65	18.90	10.25	5401.50	0.099	Covid 19	118	10.55	8.40	15.75	7.35		
Alanine transaminase IU/L	Control	105	22.00	14.50	41.50	27.00	4850.00	0.005^*^	Covid 19	118	32.50	18.00	65.00	47.00		
Alkaline phosphatase IU/L	Control	105	89.00	70.50	121.50	51.00	4975.50	0.011^*^	Covid 19	118	76.50	60.00	100.00	40.00		

* Significant *p* < 0.05. U Mann–Whitney *U* test; IQR, interquartile range; GAD-7, Generalized Anxiety Disorder-7.

### 3.2. Clinical variable

In terms of the clinical parameters, there were more patients in the COVID-19 group with respiratory symptoms (*p* = 0.001), gastrointestinal symptoms (*p* = 0.02), neurological symptoms (*p* = 0.008), and musculoskeletal symptoms (muscle and joint pain; *p* = 0.001) compared to controls.

### 3.3. Anxiety variable

The median (IQR) GAD score was significantly higher in the COVID-19 group, 8 (7,14) compared to the control group, 1 (0,2; *p* = 0.001). The proportion of the COVID-19 group who had anxiety was significantly higher (103, 86.6%) compared to the control group (16,13.4%; *p* = 0.001). The COVID-19 group had a significant association with the GAD-7 severity (*p* = 0.001). The number of COVID-19 patients in the mild, moderate, and severe anxiety groups was 48 (84.2%), 37 (86%), and 18 (94.7%), respectively. In comparison, the proportion of the control group who were in the mild, moderate, and severe categories were 9 (15.8%), 6 (14%), and 1 (5.3%), respectively.

In the laboratory parameters, the COVID-19 group had a significant association with hemoglobin (*p* = 0.04), white cell count levels (*p* = 0.005), and alanine transaminase level (*p* = 0.005; Table 1).

Table 3 shows the association between the variables and anxiety. Among the demographic factors, only employment (*p* = 0.001) and COVID-19 (*p* = 0.001) diagnoses had a significant association with anxiety. Those employed in the COVID-19 group were higher (57, 67.1%) compared to the control group (15, 12.7%). The proportion of COVID-19 patients with anxiety was higher (103, 87.3%) than the control group (15, 12.7%).

**Table 3:** Distribution of the demographic, clinical characteristics, and laboratory investigations of the study population with anxiety.

			GAD7				Total	Control	COVID-19				*n* (%)	*n* (%)	*χ* ^2^	*p*
*Demographic variable*
Age group (years)	15–64	157	71 (45.2)	86 (54.8)	0.43	0.514	> 65	66	33 (50)	33 (50)		
Gender	Male	141	67 (47.5)	74 (52.5)	0.12	0.729	Female	82	37 (45.1)	45 (54.9)		
Marital status	Single	41	20 (48.8)	21 (51.2)	0.09	0.761	Married	182	84 (46.2)	98 (53.8)		
Ethnic group	Malay	138	66 (47.8)	72 (52.2)	1.31	0.727	Chinese	52	22 (42.3)	30 (57.7)			Indian	18	10 (55.6)	8 (44.4)			Others	15	6 (40)	9 (60)		
Habits	None	182	89 (48.9)	93 (51.1)	6.58	0.087	Smoking	34	15 (44.1)	19 (55.9)			Alcohol	2	0 (0)	2 (100)			Smoking and Alcohol	5	0 (0)	5 (100)		
Education level	None	6	3 (50)	3 (50)	2.75	0.432	Primary	58	31 (53.4)	27 (46.6)			Secondary	133	56 (42.1)	77 (57.9)			University	26	14 (53.8)	12 (46.2)		
Employment	Unemployed	138	76 (55.1)	62 (44.9)	10.35	0.001^*^	Employed	85	28 (32.9)	57 (67.1)		
Diagnosis of COVID-19	No	105	89 (84.8)	16 (15.2)	115.90	0.001^*^	Yes	118	15 (12.7)	103 (87.3)		
COVID-19 category	Non-COVID	105	89 (84.8)	16 (15.2)	116.74	0.001^*^	Category 1	8	2 (25)	6 (75)			Category 2	16	2 (12.5)	14 (87.5)			Category 3	26	2 (7.7)	24 (92.3)			Category 4	57	8 (14)	49 (86)			Category 5	11	1 (9.1)	10 (90.9)		
*Clinical characteristics*
Respiratory symptoms (fever, runny nose, sore throat, shortness of breath, and cough)	No	57	39 (68.4)	18 (31.6)	14.60	0.001^*^	Yes	166	65 (39.2)	101 (60.8)		
Fever	No	99	55 (55.6)	44 (44.4)	5.69	0.017^*^	Yes	124	49 (39.5)	75 (60.5)		
Runny nose	No	205	103 (50.2)	102 (49.8)	11.54	0.001^*^	Yes	18	1 (5.6)	17 (94.4)		
Sore throat	No	193	101 (52.3)	92 (47.7)	17.03	0.001^*^	Yes	30	3 (10)	27 (90)		
Shortness of breath	No	155	83 (53.5)	72 (46.5)	8.87	0.003^*^	Yes	68	21 (30.9)	47 (69.1)		
Cough	No	134	85 (63.4)	49 (36.6)	38.06	0.001^*^	Yes	89	19 (21.3)	70 (78.7)		
Gastrointestinal symptoms (diarrhea, vomiting, abdominal pain, and poor oral intake)	No	207	91 (44)	116 (56)	6.87	0.009^*^	Yes	16	13 (81.3)	3 (18.8)		
Diarrhea	No	218	102 (46.8)	116 (53.2)	0.00	1.000	Yes	5	2 (40)	3 (60)		
Vomiting	No	220	101 (45.9)	119 (54.1)	1.65	0.200	Yes	3	3 (100)	0 (0)		
Abdominal pain	No	216	98 (45.4)	118 (54.6)	2.96	0.085	Yes	7	6 (85.7)	1 (14.3)		
Poor oral intake	No	219	100 (45.7)	119 (54.3)	2.73	0.098	Yes	4	4 (100)	0 (0)		
Neurological symptoms (seizures, weakness, headache, and dizziness)	No	181	94 (51.9)	87 (48.1)	10.84	0.001^*^	Yes	42	10 (23.8)	32 (76.2)		
Seizures	No	220	102 (46.4)	118 (53.6)	0.01	0.906	Yes	3	2 (66.7)	1 (33.3)		
Weakness	No	217	102 (47)	115 (53)	0.06	0.805	Yes	6	2 (33.3)	4 (66.7)		
Headache	No	197	101 (51.3)	96 (48.7)	14.57	0.001^*^	Yes	26	3 (11.5)	23 (88.5)		
Dizziness	No	212	100 (47.2)	112 (52.8)	0.49	0.484	Yes	11	4 (36.4)	7 (63.6)		
Muscle and joint pain	No	206	102 (49.5)	104 (50.5)	8.99	0.003^*^	Yes	17	2 (11.8)	15 (88.2)		
*Laboratory investigations*
Hemoglobin g/dL	12.0–15.0	99	43 (43.4)	56 (56.6)	0.73	0.392	Abnormal	124	61 (49.2)	63 (50.8)		
White cell count × 10^9^/L	4.0–10.0	124	53 (42.7)	71 (57.3)	1.70	0.192	Abnormal	99	51 (51.5)	48 (48.5)		
Platelet × 10^9^/L	150–410	167	78 (46.7)	89 (53.3)	0.00	0.971	Abnormal	56	26 (46.4)	30 (53.6)		
Sodium mmol/L	136–145	115	61 (53)	54 (47)	3.92	0.048^*^	Abnormal	108	43 (39.8)	65 (60.2)		
Potassium mmol/L	3.5–5.1	176	87 (49.4)	89 (50.6)	2.62	0.105	Abnormal	47	17 (36.2)	30 (63.8)		
Urea mmol/L	2.5–6.7	129	59 (45.7)	70 (54.3)	0.10	0.752	Abnormal	94	45 (47.9)	49 (52.1)		
Creatinine μmol/L	50.4–98.1	124	53 (42.7)	71 (57.3)	1.70	0.192	Abnormal	99	51 (51.5)	48 (48.5)		
Total protein g/L	64–83	170	75 (44.1)	95 (55.9)	1.82	0.177	Abnormal	53	29 (54.7)	24 (45.3)		
Albumin g/L	34–48	106	47 (44.3)	59 (55.7)	0.43	0.513	Abnormal	117	57 (48.7)	60 (51.3)		
Bilirubin μmol/L	3.4–20.5	183	88 (48.1)	95 (51.9)	0.86	0.353	Abnormal	40	16 (40)	24 (60)		
Alanine transaminase IU/L	0–55	180	88 (48.9)	92 (51.1)	1.90	0.168	Abnormal	43	16 (37.2)	27 (62.8)		
Alkaline phosphatase IU/L	40–150	191	90 (47.1)	101 (52.9)	0.13	0.724	Abnormal	32	14 (43.8)	18 (56.3)		

^*^Significant *p* < 0.05; *χ*^2^ Chi-square test.

The proportion of COVID-19 patients with anxiety was higher (103, 87.3%) compared to the control group (15, 12.7%; *p* = 0.001). Among the COVID-19 categories, category 3 had the highest proportion of patients (24, 92.3%) with anxiety (*p* = 0.001). The clinical symptoms that had a significant association with anxiety were respiratory symptoms (*p* = 0.001), neurological symptoms (*p* = 0.001), and musculoskeletal symptoms (*p* = 0.003). Among the investigations, only sodium level was associated with anxiety, where the number of COVID-19 patients with low sodium levels was 65(60.2%) compared to 43 (47.6%) in the control group (*p* = 0.048).

### 3.4. Risk factors for anxiety

Results of the univariate and multiple logistic regression analysis are shown in Table 4. In the univariate analysis, several factors showed significance for anxiety. These factors include the presence of diagnosis, employment, diabetes mellitus, respiratory symptoms, neurological symptoms, gastrointestinal symptoms, and musculoskeletal symptoms (*p* < 0.05). Further analysis by multiple logistic regression showed significant predictors for anxiety, including COVID-19 diagnosis and neurological symptoms. The diagnosis of COVID-19 was more likely to have anxiety compared to non-COVID-19 diseases (OR 36.92; 95% CI 17.09, 79.78, *p* = 0.001). We also found that patients with neurological symptoms were 2.94 times likely to have anxiety (OR 2.94; 95% CI 1.03, 8.41, *p* = 0.044; Table 4).

**Table 4:** Univariate and multivariate logistic regression analysis for anxiety.

Variables		Univariate			Multivariate	
	OR	95% CI for EXP (B)	*p*	OR	95% CI for EXP (B)	*p*
Diabetes mellitus	0.51	0.29–0.88	0.015 ^*^	0.71	0.30–1.68	0.434
Employment	2.50	1.42–4.38	0.001 ^*^	1.86	0.75–4.61	0.178
COVID-19 diagnosis	38.20	17.87–81.62	0.001 ^*^	36.92	17.09–79.78	0.001^*^
Respiratory symptoms	3.37	1.78–6.38	0.001 ^*^	1.74	0.34–8.77	0.504
Fever	1.91	1.12–3.27	0.018 ^*^	0.40	0.12–1.37	0.144
Cough	6.39	3.45–11.84	0.001 ^*^	1.74	0.63–4.77	0.284
Sore throat	9.88	2.90–33.66	0.001 ^*^	0.59	0.11–3.04	0.526
Shortness of breath	2.58	1.41–4.72	0.002 ^*^	0.54	0.18–1.63	0.275
Runny nose	17.17	2.24–131.40	0.006 ^*^	2.193	0.22–22.20	0.506
Neurological symptoms	3.46	1.60–7.45	0.002 ^*^	2.94	1.03–8.41	0.044 ^*^
Headache	8.07	2.35–27.74	0.001 ^*^	0.52	0.06–4.87	0.566
Gastrointestinal symptoms	0.18	0.05–0.65	0.009 ^*^	0.24	0.04–1.56	0.137
Muscle and joint pain	7.36	1.64–32.98	0.009 ^*^	1.40	0.17–11.16	0.753
Sodium	0.59	0.34–1.00	0.049 ^*^	1.66	0.72–3.84	0.239

^*^Significant *p* < 0.05; OR, odds ratio; 95% CI, 95% confidence interval.

## 4. Discussion

### 4.1. Prevalence of anxiety

The impact of the COVID-19 pandemic has caused a resultant increase in the psychological burden, including anxiety disorders. Mental health issues have emerged in general society but have also affected hospitalized patients (20, 21). The reported prevalence of anxiety in hospitalized COVID-19 patients ranged from 34.72% (2) to 60.35% (20, 21). A study performed in an urban hospital in Bangladesh showed that 30.7% of hospitalized patients with anxiety (22). A systemic review found that patients experience symptoms of anxiety (30–39%), depression (9–26%), and insomnia (24–40%) during and 3 months post-COVID-19 hospitalization (23).

A study from a local hospital in Malaysia regarding the psychological impact of COVID-19 patients found that the proportions of depressed, moderately anxious, and stressed patients were 20.5, 38.9, and 17.3%, respectively (24). This study was carried out in Ipoh, the capital of the Malaysian state of Perak, which is situated about 180 km north of Kuala Lumpur, the capital of Malaysia. Another local study reported a prevalence rate of 7% among stable hospitalized patients (25). On the contrary, our data from an urban tertiary teaching hospital in Kuala Lumpur revealed a higher prevalence of anxiety in hospitalized COVID-19 patients at 86.6%. The presence of COVID-19 has a 36 higher odds ratio to developing anxiety. This is in keeping with the predicted increment in anxiety disorders, posttraumatic stress disorders, obsessive–compulsive disorders, and the aversive social effects of isolation in Malaysia (26). An earlier community survey of anxiety in 2015, before the COVID-19 pandemic, only showed a prevalence of 8.2% in Malaysia (27). Following the pandemic, the prevalence of depression and anxiety is higher in the urban population compared with the rural population in Malaysia. The proportion of the participants with depressive symptoms was 23.9%; anxiety symptoms, 41.7%; and depression with comorbid anxiety symptoms, 19.9% (13). The discrepancy in the prevalence of anxiety in hospitalized COVID-19 patients between our study and other studies may be attributed to the emergence of psychosocial health problems in a middle-income country. This is supported by the reports emphasizing that the lack of financial and health resources and overcrowding may contribute to more dire consequences in low- and middle-income countries (28).

Anxiety was found to be associated with the severity of COVID-19 in the study, where the prevalence of mild, moderate, and severe anxiety was 84.2, 86, and 94.7%, respectively. This finding was in line with a previous study of hospitalized patients with severe and very severe anxiety (14). Signs and symptoms of anxiety and depression, such as irritability, despair, abnormally low mood, and discomfort, were demonstrated by COVID-19 patients in isolation wards (29). This is invariably evident in the increased vulnerability to stress and negative emotions from confined conditions and social isolation. Earlier studies from Wuhan, China, revealed that patients with low oxygen saturation related to the severe COVID-19 category were likely to have higher anxiety scores (2). There was a significant association between the severity of COVID-19 infection with anxiety in this study, whereby category 3 had the highest proportion of anxiety (92.3%) followed by category 5 (90.9%) and category 2 (87.5%), respectively. Although this study did not specifically ascertain the level of oxygen saturation during the study recruitment, the category of the patient’s severity was a more objective determinant as oxygen saturation may show a variable fluctuation during the course of the hospitalization.

### 4.2. Risk factors for anxiety

Anxiety symptoms result in clinically significant distress in the social and occupational life domains. Thus, it was not surprising that this study found that employment status was significantly different between the COVID-19 group and the controls, whereby the employed group had higher anxiety levels. Employment is crucial for psychological wellbeing as it fulfills essential needs such as social support, self-development, self-efficacy, and quality of life (30). Psychological health analysis among Chinese employees following the COVID-19 outbreak found a positive and significant impact of job insecurity on depression and anxiety (30). A cross-sectional online survey found that about 50.5% of Japanese workers felt anxious about being infected with COVID-19 in the workplace (31). A similar pattern of work-related distress was reported by employees in Serbia, where 63.4% of participants expressed increased levels of distress. This was related to moderately or highly insecure employment (30.4%) and losing their jobs (15.1%) (32). Higher distress scores were seen with increasing job insecurity, intolerance of uncertainty, and fear of COVID-19. A study in the United States gleaning the mental health burden among young adults found that job insecurity stemming from the loss of jobs and expected job loss could increase symptoms of anxiety and depression (33). The relationship between the effects of COVID-19 on the impact on employment invariably leads to poorer mental health worldwide. Further analysis of the subtypes of employment in this study may elucidate the moderating effect of intolerance of uncertainty on individual psychological factors.

Although most of the clinical features of COVID-19 are respiratory, cardiac, or gastrointestinal, many patients also experience neuropsychiatric manifestations. These manifestations stem from the direct effects on the nervous system or para-infectious/postinfectious immune-mediated disorders. Psychological stressors occur from social isolation, fear of illness, stigma, and future uncertainty from the disease. Several postulated mechanisms that have been proposed for nervous system damage by SARS-CoV-2 infection include direct infection (34), hypoxia injury (35), immune injury (36), and interaction with the angiotensin-converting enzyme receptors (37).

Several papers have explored the various COVID-19 neurological manifestations from China (38), ALBACOVID in Spain (39), the United States (40), France (41), and Malaysia (42). A systemic review of the literature revealed common neurological manifestations: myalgia, taste impairment, smell impairment, headache, and dizziness (43, 44). More severe complications include encephalopathy, encephalitis, cerebrovascular diseases (41, 45) and Guillain–Barre syndrome (46). Mao et al. retrospectively analyzed COVID-19 patients from 3 hospitals (38). They found 36.4% of patients with neuropsychiatric symptoms, which were differentiated into central (dizziness, headache), peripheral (dysgeusia, anosmia, and muscle pain), and psychological (anxiety, depression, and delirium) (38). Similarly, this study revealed that the main neurological symptoms were seizures, weakness, headache, and dizziness.

Previous literature on mental health in COVID-19 was primarily derived from observational studies (5, 47). The common psychological reactions to the COVID-19 pandemic showed symptoms of anxiety and depression (16–28%) and self-reported stress (8%) and may be associated with disturbed sleep (48). In this study, we evaluated that neurological symptoms had almost thrice the odds of developing anxiety symptoms. The currently available data broadly describe the neuropsychological COVID-19 manifestations but do not explore the association between both aspects. Our study demonstrated the possibility that anxiety might also be likely related to the underlying complex interplay of neurological features. Several proposed mechanisms that interlink psychopathological factors and immune systems include neuronal injury (49), disruption of the blood–brain barrier, peripheral immune cell invasion into the central nervous system (50) and maladaptive immune systems ( 51).

Anxiety is often associated with negative outcomes such as poorer prognosis of physical diseases, longer hospitalization, and increased readmission rates in non-psychiatric settings (52). The consequences of anxiety may affect the quality of life of the individual and negatively affect the individual’s work, family, and social life, and even lead to suicide (53). The effects of anxiety are often seen in isolation and quarantine wards. The unfavorable psychological effects of quarantine may lead to post-traumatic stress symptoms, bewilderment, and rage (54). The impact of the pandemic on anxiety needs to be apprehended in order to tailor the appropriate psychological and social support.

### 4.3. Strengths and limitations

The case–control study measured the variables between the cases and controls to evaluate the significant risk factors. We identified that COVID-19 patients with neurological symptoms had a higher risk to develop anxiety, which is a novel finding.

This study was carried out at a single center, which may not be a representative of the wider population of COVID-19 patients. This significant limitation may underestimate the true prevalence of anxiety among this patient group. Moreover, the study cannot determine the temporal relationship between exposure and outcome, which is a key consideration in understanding the development of anxiety. The lack of follow-up of patients after discharge from the hospital precludes any assessment of whether anxiety levels persist over time. The use of only one anxiety assessment questionnaire limits the ability to compare anxiety levels with other validated tools.

## 5. Conclusion

This study compared patients’ characteristics, clinical features, and anxiety levels concerning COVID-19 patients compared to controls from a tertiary teaching hospital setting. We identified that the burden of anxiety is high among hospitalized COVID-19 patients compared to controls. Those with the presence of neurological symptoms were more likely to suffer from anxiety. Early psychiatric referrals are warranted for patients at risk of having symptoms of anxiety. In addition, the availability of support groups to provide counseling assistance to hospitalized COVID-19 patients may help to facilitate support intervention programs.

## Data availability statement

The original contributions presented in the study are included in the article/Supplementary material, further inquiries can be directed to the corresponding author.

## Ethics statement

The studies involving human participants were reviewed and approved by Research and Ethics Board, Faculty of Medicine, National University of Malaysia. The patients/participants provided their written informed consent to participate in this study.

## Author contributions

AS and HT: conceptualization, methodology, investigation, formal analysis, and writing—original draft. CK: investigation, formal analysis, and writing—original draft. CN: methodology, investigation, formal analysis, and writing—review and editing. WZ: methodology, investigation, and writing—review and editing. NK and PP: data acquisition, formal analysis, and writing—review and editing. CE and AP: data acquisition and writing—review and editing. RH and HT: methodology and writing—review and editing. All authors contributed to the article and approved the submitted version.

## Funding

This study was funded by the National University of Malaysia Research Grant (FF-2021-379).

## Conflict of interest

The authors declare that the research was conducted without any commercial or financial relationships that could be construed as a potential conflict of interest.

## Publisher’s note

All claims expressed in this article are solely those of the authors and do not necessarily represent those of their affiliated organizations, or those of the publisher, the editors and the reviewers. Any product that may be evaluated in this article, or claim that may be made by its manufacturer, is not guaranteed or endorsed by the publisher.

## Abbreviations

COVID-19: Coronavirus disease 2019
GAD-7: General Anxiety Disorder-7
SARS-CoV-2: Severe Acute Respiratory Syndrome Coronavirus 2
MERS-CoV: Middle East respiratory syndrome–related coronavirus

## References

[1] de Sousa GM Tavares VDO de Meiroz Grilo MLP Coelho MLG Lima-Araújo GL Schuch FB. Mental health in COVID-19 pandemic: a meta-review of prevalence Meta-analyses. Front Psychol. (2021) 12:703838. doi: 10.3389/fpsyg.2021.703838 34621212 PMC8490780

[2] Dai LL Wang X Jiang TC Li PF Wang Y Wu SJ. Anxiety and depressive symptoms among COVID-19 patients in Jianghan Fangcang shelter Hospital in Wuhan, China. PLoS One. (2020) 15:15. doi: 10.1371/journal.pone.0238416 PMC7454940 32857826

[3] Kong X Zheng K Tang M Kong F Zhou J Diao L. (2020). Prevalence and factors associated with depression and anxiety of hospitalized patients with COVID-19. medRxiv 2020.03.24.20043075; doi: 10.1101/2020.03.24.20043075 [Epub ahead of print].

[4] Tokur Kesgin M Hançer Tok H Uzun LN Pehlivan Ş. Comparison of anxiety levels of hospitalized COVID-19 patients, individuals under quarantine, and individuals in society. Perspect Psychiatr Care. (2022) 58:149–58. doi: 10.1111/ppc.12857, PMID: 34018193 PMC8242866

[5] Zandifar A Badrfam R Yazdani S Arzaghi SM Rahimi F Ghasemi S. Prevalence and severity of depression, anxiety, stress and perceived stress in hospitalized patients with COVID-19. J Diabetes Metab Disord. (2020) 19:1431–8. doi: 10.1007/s40200-020-00667-1, PMID: 33145259 PMC7594988

[6] Lancet Regional Health–Southeast Asia T. New vision for a healthier Southeast Asia-NC-ND license. Lancet Regional Health–Southeast Asia. (2022) 1:100020. doi: 10.1016/j.lansea.2022.100020 PMC10305909 37383095

[7] Lim PY Md Said S Kadir Shahar H Azman AZF Mokhtar SA Mahmud A. COVID-19 inpatient deaths and brought-in-dead cases in Malaysia. Front Public Health. (2022) 10:10. doi: 10.3389/fpubh.2022.872838 PMC9298663 35875031

[8] Narendra Kumar MK Francis B Hashim AH Zainal NZ Abdul Rashid R Ng CG. Prevalence of anxiety and depression among psychiatric healthcare workers during the COVID-19 pandemic: a Malaysian perspective. Healthcare (Basel). (2022) 10:532–47. doi: 10.3390/healthcare10030532 35327009 PMC8951112

[9] Nordin S Yaacob NA Kelak J Ilyas AH Daud A. The mental health of Malaysia’s northwest healthcare workers during the relaxation of COVID-19 restrictions and its associated factors. Int J Environ Res Public Health. (2022) 19:7794–7803. doi: 10.3390/ijerph19137794 35805454 PMC9266069

[10] Fong HX Cornish K Kirk H Ilias K Shaikh MF Golden KJ. Impact of the COVID-19 lockdown in Malaysia: An examination of the psychological well-being of parent-child dyads and child behavior in families with children on the autism spectrum. Front Psych. (2021) 12:12. doi: 10.3389/fpsyt.2021.733905 PMC8555492 34721108

[11] Abdul Latif NI Mohamed Ismail NA Loh SYE Nur Azurah AG Midin M Shah SA. Psychological distress and COVID-19 related anxiety among Malaysian women during the COVID-19 pandemic. Int J Environ Res Public Health. (2022) 1:19. doi: 10.3390/ijerph19084590 PMC9024897 35457456

[12] Marzo RR Vinay V Bahari R Chauhan S Ming DAF Nelson Fernandez SFA/P. Depression and anxiety in Malaysian population during third wave of the COVID-19 pandemic. Clin Epidemiol Glob Health. (2021) 12:100868. doi: 10.1016/j.cegh.2021.100868 34549098 PMC8444444

[13] Leong Bin Abdullah MFI Ahmad Yusof H Mohd Shariff N Hami R Nisman NF Law KS. Depression and anxiety in the Malaysian urban population and their association with demographic characteristics, quality of life, and the emergence of the COVID-19 pandemic. Curr Psychol. (2021) 40:6259–70. doi: 10.1007/s12144-021-01492-2, PMID: 33623353 PMC7892323

[14] Woon LSC Leong Bin Abdullah MFI Sidi H Mansor NS Nik Jaafar NR. Depression, anxiety, and the COVID-19 pandemic: severity of symptoms and associated factors among university students after the end of the movement lockdown. PLoS One. (2021) 16:16. doi: 10.1371/journal.pone.0252481 PMC8158968 34043731

[15] Ministry of Health Malaysia. Clinical management of confirmed COVID-19 case in adult. Ministry of Health Malaysia. (2020). Available at: https://covid-19.moh.gov.my/garis-panduan/gp-umum-covid19 (Accessed Dec 20, 2022).

[16] Spitzer RL Kroenke K Williams JB Löwe B. A brief measure for assessing generalized anxiety disorder: the GAD-7. Arch Intern Med. (2006) 166:1092–7. doi: 10.1001/archinte.166.10.1092, PMID: 16717171

[17] Sidik SM Arroll B Goodyear-Smith F. Validation of the GAD-7 (Malay version) among women attending a primary care clinic in Malaysia. J Prim Health Care. (2012) 4:5–11. doi: 10.1071/HC12005, PMID: 22377544

[18] Cordaro M Grigsby TJ Howard JT Deason RG Haskard-Zolnierek K Howard K. Pandemic-specific factors related to generalized anxiety disorder during the initial COVID-19 protocols in the United States. Issues Ment Health Nurs. (2021) 42:747–57. doi: 10.1080/01612840.2020.1867675, PMID: 33480832

[19] Chen H Gao J Dai J Mao Y Wang Y Chen S. Generalized anxiety disorder and resilience during the COVID-19 pandemic: evidence from China during the early rapid outbreak. BMC Public Health. (2021) 21:1830. doi: 10.1186/s12889-021-11877-4 34627208 PMC8502085

[20] Hao F Tam W Hu X Tan W Jiang L Jiang X. A quantitative and qualitative study on the neuropsychiatric sequelae of acutely ill COVID-19 inpatients in isolation facilities. Transl Psychiatry. (2020) 10:355. doi: 10.1038/s41398-020-01039-2, PMID: 33077738 PMC7570419

[21] Ngasa SN Tchouda LAS Abanda C Ngasa NC Sanji EW Dingana TN. Prevalence and factors associated with anxiety and depression amongst hospitalised COVID-19 patients in Laquintinie hospital Douala, Cameroon. PLoS One. (2021) 16:e0260819. doi: 10.1371/journal.pone.0260819, PMID: 34855877 PMC8638855

[22] Nabi SG Rashid MU Sagar SK Ghosh P Shahin M Afroz F. Psychological impact of COVID-19 pandemic: a cross-sectional study of hospitalized COVID-19 patients in an urban setting, Bangladesh. Heliyon. (2022) 8:e09110. doi: 10.1016/j.heliyon.2022.e09110, PMID: 35299601 PMC8916985

[23] Veazie S Lafavor B Vela K Young S Sayer NA Carlson KF. Mental health outcomes of adults hospitalized for COVID-19: a systematic review. J Affect Disord Rep. (2022) 8:100312. doi: 10.1016/j.jadr.2022.100312, PMID: 35165670 PMC8828444

[24] Loo TH Arvinder-Singh HS Ang YC Kong YH Vikram Suarn S Rakesh S. Psychological impact amongst patients with COVID-19 in Perak state. Med J Malaysia. (2022) 77:643–9. PMID: 36448379

[25] Bin Adnan MAA Bin Kassim MSA Bt Sahril N. Prevalence and predictors of anxiety among stable hospitalized COVID-19 patients in Malaysia. Int J Environ Res Public Health. (2023) 20:586. doi: 10.3390/ijerph20010586 PMC9819532 36612905

[26] Shanmugam H Ariff JJ Nair P Ken CS Guan NC. Impacts of COVID-19 pandemic on mental health in Malaysia: a single thread of hope. Malays J Psychiatry. (2020) 29:78–84.

[27] Maideen SFK Sidik SM Rampal L Mukhtar F. Prevalence, associated factors and predictors of anxiety: a community survey in Selangor. Malaysia BMC Psychiatry. (2015) 15:262–273. doi: 10.1186/s12888-015-0648-x 26497745 PMC4620008

[28] Bong CL Brasher C Chikumba E McDougall R Mellin-Olsen J Enright A. The COVID-19 pandemic: effects on low- and middle-income countries. Anesth Analg. (2020) 131:86–92. doi: 10.1213/ANE.0000000000004846, PMID: 32243287 PMC7173081

[29] Rahman J Muralidharan A Quazi SJ Saleem H Khan S. Neurological and psychological effects of coronavirus (COVID-19): An overview of the current era pandemic. Cureus. (2020) 12:e8460. doi: 10.7759/cureus.8460, PMID: 32528783 PMC7282368

[30] Khudaykulov A Changjun Z Obrenovic B Godinic D Alsharif HZH Jakhongirov I. The fear of COVID-19 and job insecurity impact on depression and anxiety: An empirical study in China in the COVID-19 pandemic aftermath. Curr Psychol. (2022) 9:1–14. doi: 10.1007/s12144-022-02883-9, PMID: 35287294 PMC8906526

[31] Eguchi H Hino A Inoue A Tsuji M Tateishi S Ando H. Effect of anxiety about COVID-19 infection in the workplace on the association between job demands and psychological distress. Front Public Health. (2021) 9:722071. doi: 10.3389/fpubh.2021.722071, PMID: 34722436 PMC8548681

[32] Blanuša J Barzut V Knežević J. Intolerance of uncertainty and fear of COVID-19 moderating role in relationship between job insecurity and work-related distress in the republic of Serbia. Front Psychol. (2021) 12:647972. doi: 10.3389/fpsyg.2021.647972 34177703 PMC8226083

[33] Ganson KT Tsai AC Weiser SD Benabou SE Nagata JM. Job insecurity and symptoms of anxiety and depression among U.S. Young adults during COVID-19. J Adolesc Health. (2021) 68:53–6. doi: 10.1016/j.jadohealth.2020.10.008, PMID: 33183926

[34] Leber AL Everhart K Balada-Llasat JM Cullison J Daly J Holt S. Multicenter evaluation of biofire filmarray meningitis/encephalitis panel for detection of bacteria, viruses, and yeast in cerebrospinal fluid specimens. J Clin Microbiol. (2016) 54:2251–61. doi: 10.1128/JCM.00730-16, PMID: 27335149 PMC5005480

[35] Guo YR Cao QD Hong ZS Tan YY Chen SD Jin HJ. The origin, transmission and clinical therapies on coronavirus disease 2019 (COVID-19) outbreak - an update on the status. Mil Med Res. (2020) 7:11. doi: 10.1186/s40779-020-00240-0, PMID: 32169119 PMC7068984

[36] Klein RS Garber C Howard N. Infectious immunity in the central nervous system and brain function. Nat Immunol Nat Publish Group. (2017) 18:132–41. doi: 10.1038/ni.3656 PMC5815515 28092376

[37] Wrapp D Wang N Corbett KS Goldsmith JA Hsieh CL Abiona O. Cryo-EM structure of the 2019-nCoV spike in the prefusion conformation. Science. (2020) 367:1260–3. doi: 10.1126/science.abb2507, PMID: 32075877 PMC7164637

[38] Mao L Jin H Wang M Hu Y Chen S He Q. Neurologic manifestations of hospitalized patients with coronavirus disease 2019 in Wuhan, China. Arch Neurol. (2020) 77:683–90. doi: 10.1001/jamaneurol.2020.1127, PMID: 32275288 PMC7149362

[39] Romero-Sánchez CM Díaz-Maroto I Fernández-Díaz E Sánchez-Larsen Á Layos-Romero A García-García J. Neurologic manifestations in hospitalized patients with COVID-19: the ALBACOVID registry. Neurology. (2020) 95:E1060–70. doi: 10.1212/WNL.0000000000009937, PMID: 32482845 PMC7668545

[40] Iosifescu AL Hoogenboom WS Buczek AJ Fleysher R Duong TQ. New-onset and persistent neurological and psychiatric sequelae of COVID-19 compared to influenza: a retrospective cohort study in a large new York City healthcare network. Int J Methods Psychiatr Res. (2022) 31:e1914. doi: 10.1002/mpr.1914, PMID: 35706352 PMC9349863

[41] Meppiel E Peiffer-Smadja N Maury A Bekri I Delorme C Desestret V. Neurologic manifestations associated with COVID-19: a multicentre registry. Clin Microbiol Infect. (2021) 27:458–66. doi: 10.1016/j.cmi.2020.11.005, PMID: 33189873 PMC7661948

[42] Tan HJ Goh CH Khoo CS Ng CF Tan JK Wan Zaidi WA. Neurological manifestations in SARS-CoV -2 infection: a single-center cross-sectional study in Malaysia. Neurol Clin Neurosci. (2023) 11:17–26. doi: 10.1111/ncn3.12677, PMID: 36714457 PMC9874463

[43] Yassin A Nawaiseh M Shaban A Alsherbini K el-Salem K Soudah O. Neurological manifestations and complications of coronavirus disease 2019 (COVID-19): a systematic review and meta-analysis. BMC Neurol. (2021) 21:138. doi: 10.1186/s12883-021-02161-4, PMID: 33784985 PMC8007661

[44] Pinzon RT Wijaya VO Buana RB al Jody A Nunsio PN. Neurologic characteristics in coronavirus disease 2019 (COVID-19): a systematic review and meta-analysis. Front Neurol. (2020) 11:565. doi: 10.3389/fneur.2020.00565, PMID: 32574250 PMC7273516

[45] Ellul MA Benjamin L Singh B Lant S Michael BD Easton A. Neurological associations of COVID-19. Lancet Neurol. (2020) 19:767–83. doi: 10.1016/S1474-4422(20)30221-0, PMID: 32622375 PMC7332267

[46] Payus AO Jan TH Ibrahim A. Autoimmune polyradiculopathy in SARS-CoV-2: a narrative review of Guillain-Barre syndrome in novel coronavirus disease (COVID-19). Acta Med Mediterranea. (2020) 36:3781–5. doi: 10.19193/0393-6384_2020_6_599

[47] Liu S Yang L Zhang C Xiang YT Liu Z Hu S. Online mental health services in China during the COVID-19 outbreak. Lancet Psychiatry. (2020) 7:e17–8. doi: 10.1016/S2215-0366(20)30077-8, PMID: 32085841 PMC7129099

[48] Rajkumar RP. COVID-19 and mental health: a review of the existing literature. Asian J Psychiatr. (2020) 52:102066. doi: 10.1016/j.ajp.2020.102066, PMID: 32302935 PMC7151415

[49] Desforges M le Coupanec A Dubeau P Bourgouin A Lajoie L Dubé M. Human coronaviruses and other respiratory viruses: underestimated opportunistic pathogens of the central nervous system? Viruses. (2019) 12:14. doi: 10.3390/v12010014, PMID: 31861926 PMC7020001

[50] Wu Y Xu X Chen Z Duan J Hashimoto K Yang L. Nervous system involvement after infection with COVID-19 and other coronaviruses. Brain Behav Immun. (2020) 87:18–22. doi: 10.1016/j.bbi.2020.03.031, PMID: 32240762 PMC7146689

[51] Najjar S Pearlman DM Alper K Najjar A Devinsky O. Neuroinflammation and psychiatric illness. J Neuroinflammation. (2013) 10:43. doi: 10.1186/1742-2094-10-43, PMID: 23547920 PMC3626880

[52] Guo WJ Wang HY Deng W Huang MJ Dong ZQ Liu Y. Effects of anxiety and depression and early detection and management of emotional distress on length of stay in hospital in non-psychiatric inpatients in China: a hospital-based cohort study. Lancet. (2019) 394:S83. doi: 10.1016/S0140-6736(19)32419-5

[53] Wang C Pan R Wan X Tan Y Xu L Ho CS. Immediate psychological responses and associated factors during the initial stage of the 2019 coronavirus disease (COVID-19) epidemic among the general population in China. Int J Environ Res Public Health. (2020) 17:1729. doi: 10.3390/ijerph17051729, PMID: 32155789 PMC7084952

[54] Brooks SK Webster RK Smith LE Woodland L Wessely S Greenberg N. The psychological impact of quarantine and how to reduce it: rapid review of the evidence. Lancet. (2020) 395:912–20. doi: 10.1016/S0140-6736(20)30460-8, PMID: 32112714 PMC7158942

