# Supplementary material for: Anxiety among hospitalized COVID-19 patients: a case–control study from a tertiary teaching hospital in Malaysia
Source: Front Psychiatry. 2023 May 18;14:1148019. doi: 10.3389/fpsyt.2023.1148019 (PMC10232945; doi:10.3389/fpsyt.2023.1148019)
Supplement: Supplementary file 1 [file Table_1.docx]

***Supplementary Material***

**Anxiety among hospitalized COVID-19 patients: a case-control study from a tertiary teaching hospital in Malaysia**

**Hui Jan Tan^1*^ , Abdool Alleem Hj Shahren ^1^, Ching Soong Khoo^1^, Chen Fei Ng^1^, Wan Asyraf Wan Zaidi^1^, Najma Kori^1^, Petrick Periasamy^1^, Choon Leng Eu^2^, Alvin Oliver Payus ^3^, Rozita Hod^4^**

^1^Department of Medicine, Faculty of Medicine, The National University of Malaysia, Kuala Lumpur, Malaysia

^2^Department of Psychiatry, Faculty of Medicine, The National University of Malaysia, Kuala Lumpur, Malaysia

^3^Department of Medicine, University Malaysia Sabah, Kota Kinabalu, Malaysia

^4^Department of Community Health, Faculty of Medicine, The National University of Malaysia, Kuala Lumpur, Malaysia

*** Correspondence**

Corresponding author: [tanhuijan@ukm.edu.my](mailto:tanhuijan@ukm.edu.my)

# Supplementary Tables

Table1 Demographic, clinical characteristics, GAD-7 score, and laboratory investigations of the study population and controls

|  |  |  |  | |  |  |
| --- | --- | --- | --- | --- | --- | --- |
|  |  |  | Control | COVID-19 |  |  |
|  |  | Total | n (%) | n (%) | χ^2^ | P |
| **Demographic variables** |  |  |  |  |  |  |
| Age group (years) | 15 – 64 | 157 | 73 (46.5) | 84 (53.5) | 0.07 | 0.786 |
|  | > 65 | 66 | 32 (48.5) | 34 (51.5) |  |  |
| Gender | Male | 141 | 68 (48.2) | 73 (51.8) | 0.20 | 0.654 |
|  | Female/ | 82 | 37 (45.1) | 45 (54.9) |  |  |
| Marital status | Single | 41 | 21 (51.2) | 20 (48.8) | 0.35 | 0.557 |
|  | Married | 182 | 84 (46.2) | 98 (53.8) |  |  |
| Ethnic group | Malay | 138 | 66 (47.8) | 72 (52.2) | 0.41 | 0.937 |
|  | Chinese | 52 | 24 (46.2) | 28 (53.8) |  |  |
|  | Indian | 18 | 9 (50) | 9 (50) |  |  |
|  | Others | 15 | 6 (40) | 9 (60) |  |  |
| Habits | None | 182 | 82 (45.1) | 100 (54.9) | 5.90 | 0.117 |
|  | Smoking | 34 | 21 (61.8) | 13 (38.2) |  |  |
|  | Alcohol | 2 | 0 (0) | 2 (100) |  |  |
|  | Smoking and Alcohol | 5 | 2 (40) | 3 (60) |  |  |
| Education level | None | 6 | 3 (50) | 3 (50) | 1.01 | 0.799 |
|  | Primary | 58 | 29 (50) | 29 (50) |  |  |
|  | Secondary | 133 | 63 (47.4) | 70 (52.6) |  |  |
|  | University | 26 | 10 (38.5) | 16 (61.5) |  |  |
| Employment | Unemployed | 138 | 77 (55.8) | 61 (44.2) | 11.03 | 0.001* |
|  | Employed | 85 | 28 (32.9) | 57 (67.1) |  |  |
| **Clinical characteristics** |  |  |  |  |  |  |
| Respiratory symptoms (fever, runny nose, sore throat, shortness of breath, cough) | No | 57 | 44 (77.2) | 13 (22.8) | 26.26 | 0.001* |
|  | Yes | 166 | 61 (36.7) | 105 (63.3) |  |  |
| Fever | No | 99 | 61 (61.6) | 38 (38.4) | 14.06 | 0.001* |
|  | Yes | 124 | 44 (35.5) | 80 (64.5) |  |  |
| Runny nose | No | 205 | 105 (51.2) | 100 (48.8) | 15.43 | 0.001* |
|  | Yes | 18 | 0 (0) | 18 (100) |  |  |
| Sore throat | No | 193 | 105 (54.4) | 88 (45.6) | 28.70 | 0.001* |
|  | Yes | 30 | 0 (0) | 30 (100) |  |  |
| Shortness of breath | No | 155 | 89 (57.4) | 66 (42.6) | 21.79 | 0.001* |
|  | Yes | 68 | 16 (23.5) | 52 (76.5) |  |  |
| Cough | No | 134 | 91 (67.9) | 43 (32.1) | 58.44 | 0.001* |
|  | Yes | 89 | 14 (15.7) | 75 (84.3) |  |  |
| Gastrointestinal symptoms (diarrhea, vomiting, abdominal pain, poor intake) | No | 207 | 93 (44.9) | 114 (55.1) | 5.39 | 0.020* |
|  | Yes | 16 | 12 (75) | 4 (25) |  |  |
| Diarrhea | No | 218 | 103 (47.2) | 115 (52.8) | 0.00 | 1.000 |
|  | Yes | 5 | 2 (40) | 3 (60) |  |  |
| Vomiting | No | 220 | 102 (46.4) | 118 (53.6) | 1.60 | 0.205 |
|  | Yes | 3 | 3 (100) | 0 (0) |  |  |
| Abdominal pain | No | 216 | 99 (45.8) | 117 (54.2) | 2.88 | 0.090 |
|  | Yes | 7 | 6 (85.7) | 1 (14.3) |  |  |
| Poor intake | No | 219 | 102 (46.6) | 117 (53.4) | 0.39 | 0.533 |
|  | Yes | 4 | 3 (75) | 1 (25) |  |  |
| Neurological symptoms (seizures, weakness, headache, dizziness) | No | 181 | 93 (51.4) | 88 (48.6) | 7.12 | 0.008* |
|  | Yes | 42 | 12 (28.6) | 30 (71.4) |  |  |
| Seizures | No | 220 | 103 (46.8) | 117 (53.2) | 0.01 | 0.919 |
|  | Yes | 3 | 2 (66.7) | 1 (33.3) |  |  |
| Weakness | No | 217 | 100 (46.1) | 117 (53.9) | 1.93 | 0.165 |
|  | Yes | 6 | 5 (83.3) | 1 (16.7) |  |  |
| Headache | No | 197 | 103 (52.3) | 94 (47.7) | 16.58 | 0.001* |
|  | Yes | 26 | 2 (7.7) | 24 (92.3) |  |  |
| Dizziness | No | 212 | 102 (48.1) | 110 (51.9) | 1.08 | 0.298 |
|  | Yes | 11 | 3 (27.3) | 8 (72.7) |  |  |
| Muscle and joint pain | No | 206 | 104 (50.5) | 102 (49.5) | 10.81 | 0.001* |
|  | Yes | 17 | 1 (5.9) | 16 (94.1) |  |  |
| **Comorbidities** |  |  |  |  |  |  |
| Diabetes mellitus | No | 139 | 58 (41.7) | 81 (58.3) | 4.25 | 0.039* |
|  | Yes | 84 | 47 (56) | 37 (44) |  |  |
| Hypertension | No | 125 | 56 (44.8) | 69 (55.2) | 0.60 | 0.440 |
|  | Yes | 98 | 49 (50) | 49 (50) |  |  |
| Chronic kidney disease | No | 193 | 87 (45.1) | 106 (54.9) | 2.32 | 0.128 |
|  | Yes | 30 | 18 (60) | 12 (40) |  |  |
| Dyslipidemia | No | 189 | 91 (48.1) | 98 (51.9) | 0.56 | 0.453 |
|  | Yes | 34 | 14 (41.2) | 20 (58.8) |  |  |
| Ischemic heart disease | No | 191 | 89 (46.6) | 102 (53.4) | 0.13 | 0.721 |
|  | Yes | 32 | 16 (50) | 16 (50) |  |  |
| Bronchial asthma | No | 208 | 102 (49) | 106 (51) | 3.64 | 0.056 |
|  | Yes | 15 | 3 (20) | 12 (80) |  |  |
| **Anxiety variable** |  |  |  |  |  |  |
| GAD-7 score | No anxiety | 104 | 89 (85.6) | 15 (14.4) | 115.90 | 0.001* |
|  | Anxiety | 119 | 16 (13.4) | 103 (86.6) |  |  |
| GAD-7 severity | No anxiety | 104 | 89 (85.6) | 15 (14.4) | 130.26 | 0.001* |
|  | Mild | 57 | 9 (15.8) | 48 (84.2) |  |  |
|  | Moderate | 43 | 6 (14) | 37 (86) |  |  |
|  | Severe | 19 | 1 (5.3) | 18 (94.7) |  |  |
| **Laboratory investigations** |  |  |  |  |  |  |
| Hemoglobin g/dL | 12.0 - 15.0 | 99 | 39 (39.4) | 60 (60.6) | 4.23 | 0.040* |
|  | Abnormal | 124 | 66 (53.2) | 58 (46.8) |  |  |
| White cell count x10^9^/L | 4.0 - 10.0 | 124 | 48 (38.7) | 76 (61.3) | 7.86 | 0.005* |
|  | Abnormal | 99 | 57 (57.6) | 42 (42.4) |  |  |
| Platelet x10^9^/L | 150 - 410 | 167 | 76 (45.5) | 91 (54.5) | 0.66 | 0.415 |
|  | Abnormal | 56 | 29 (51.8) | 27 (48.2) |  |  |
| Sodium mmol/L | 136 - 145 | 115 | 61 (53) | 54 (47) | 3.38 | 0.066 |
|  | Abnormal | 108 | 44 (40.7) | 64 (59.3) |  |  |
| Potassium mmol/L | 3.5 - 5.1 | 176 | 88 (50) | 88 (50) | 2.85 | 0.092 |
|  | Abnormal | 47 | 17 (36.2) | 30 (63.8) |  |  |
| Urea mmol/L | 2.5 - 6.7 | 129 | 57 (44.2) | 72 (55.8) | 1.03 | 0.310 |
|  | Abnormal | 94 | 48 (51.1) | 46 (48.9) |  |  |
| Creatinine µmol/L | 50.4 - 98.1 | 124 | 53 (42.7) | 71 (57.3) | 2.12 | 0.146 |
|  | Abnormal | 99 | 52 (52.5) | 47 (47.5) |  |  |
| Total protein g/L | 64 - 83 | 170 | 78 (45.9) | 92 (54.1) | 0.42 | 0.519 |
|  | Abnormal | 53 | 27 (50.9) | 26 (49.1) |  |  |
| Albumin g/L | 34 - 48 | 106 | 49 (46.2) | 57 (53.8) | 0.06 | 0.807 |
|  | Abnormal | 117 | 56 (47.9) | 61 (52.1) |  |  |
| Bilirubin µmol/L | 3.4 - 20.5 | 183 | 85 (46.4) | 98 (53.6) | 0.17 | 0.683 |
|  | Abnormal | 40 | 20 (50) | 20 (50) |  |  |
| Alanine transaminase IU/L | 0 - 55 | 180 | 93 (51.7) | 87 (48.3) | 7.86 | 0.005* |
|  | Abnormal | 43 | 12 (27.9) | 31 (72.1) |  |  |
| Alkaline phosphatase IU/L | 40 - 150 | 191 | 89 (46.6) | 102 (53.4) | 0.13 | 0.721 |
|  | Abnormal | 32 | 16 (50) | 16 (50) |  |  |

*Significant P<0.05 χ^2^ Chi-square test, GAD-7 Generalised anxiety disorder-7

Table 2 Comparison of the variables between the study population and controls

|  |  |  |  | Percentiles |  |  |  |  |
| --- | --- | --- | --- | --- | --- | --- | --- | --- |
|  | Group | N | 50th (Median) | 25th | 75th | IQR | U | P |
| Age (years) | Control | 105 | 56.00 | 37.50 | 68.00 | 30.50 | 6180.00 | 0.975 |
|  | Covid 19 | 118 | 54.00 | 40.75 | 65.00 | 24.25 |  |  |
| GAD-7 Score | Control | 105 | 1.00 | 0.00 | 2.00 | 2.00 | 1410.50 | 0.001* |
|  | Covid 19 | 118 | 8.00 | 7.00 | 14.00 | 7.00 |  |  |
| Length of stay | Control | 105 | 10.00 | 5.00 | 17.50 | 12.50 | 5163.50 | 0.032* |
|  | Covid 19 | 118 | 12.00 | 8.00 | 20.25 | 12.25 |  |  |
| Hemoglobin level (g/dL) | Control | 105 | 12.10 | 10.15 | 14.05 | 3.90 | 5062.50 | 0.019* |
|  | Covid 19 | 118 | 13.40 | 11.48 | 14.73 | 3.25 |  |  |
| White cell count x10^9^/L | Control | 105 | 10.30 | 8.05 | 15.10 | 7.05 | 4108.50 | 0.001* |
|  | Covid 19 | 118 | 8.50 | 5.98 | 10.50 | 4.53 |  |  |
| Platelet x10^9^/L | Control | 105 | 276.00 | 208.00 | 345.00 | 137.00 | 4881.50 | 0.006* |
|  | Covid 19 | 118 | 236.50 | 177.75 | 289.00 | 111.25 |  |  |
| Sodium mmol/L | Control | 105 | 136.00 | 133.00 | 139.00 | 6.00 | 5174.00 | 0.033* |
|  | Covid 19 | 118 | 135.00 | 131.00 | 138.00 | 7.00 |  |  |
| Potassium mmol/L | Control | 105 | 4.00 | 3.70 | 4.40 | 0.70 | 5264.50 | 0.053 |
|  | Covid 19 | 118 | 3.90 | 3.58 | 4.30 | 0.73 |  |  |
| Urea mmol/L | Control | 105 | 5.50 | 3.80 | 10.20 | 6.40 | 5619.00 | 0.231 |
|  | Covid 19 | 118 | 4.90 | 3.30 | 7.63 | 4.33 |  |  |
| Creatinine µmol/L | Control | 105 | 97.20 | 75.65 | 180.40 | 104.75 | 5196.00 | 0.038* |
|  | Covid 19 | 118 | 86.80 | 72.75 | 129.83 | 57.08 |  |  |
| Total protein g/L | Control | 105 | 71.00 | 65.50 | 78.00 | 12.50 | 6172.50 | 0.963 |
|  | Covid 19 | 118 | 72.50 | 66.00 | 78.00 | 12.00 |  |  |
| Albumin g/L | Control | 103 | 33.00 | 27.00 | 38.00 | 11.00 | 6031.00 | 0.923 |
|  | Covid 19 | 118 | 33.00 | 29.00 | 37.00 | 8.00 |  |  |
| Bilirubin µmol/L | Control | 105 | 12.30 | 8.65 | 18.90 | 10.25 | 5401.50 | 0.099 |
|  | Covid 19 | 118 | 10.55 | 8.40 | 15.75 | 7.35 |  |  |
| Alanine transaminase IU/L | Control | 105 | 22.00 | 14.50 | 41.50 | 27.00 | 4850.00 | 0.005* |
|  | Covid 19 | 118 | 32.50 | 18.00 | 65.00 | 47.00 |  |  |
| Alkaline phosphatase IU/L | Control | 105 | 89.00 | 70.50 | 121.50 | 51.00 | 4975.50 | 0.011* |
|  | Covid 19 | 118 | 76.50 | 60.00 | 100.00 | 40.00 |  |  |

*Significant P<0.05 U Mann-Whitney U test, IQR interquartile range, GAD-7 Generalised Anxiety Disorder-7

Table 3 Distribution of the demographic, clinical characteristics, and laboratory investigations of the study population with anxiety

|  |  |  | GAD7 | |  |  |
| --- | --- | --- | --- | --- | --- | --- |
|  |  | Total | Control | COVID-19 |  |  |
|  |  |  | n (%) | n (%) | χ^2^ | P |
| **Demographic variable** |  |  |  |  |  |  |
| Age group (years) | 15 - 64 | 157 | 71 (45.2) | 86 (54.8) | 0.43 | 0.514 |
|  | > 65 | 66 | 33 (50) | 33 (50) |  |  |
| Gender | Male | 141 | 67 (47.5) | 74 (52.5) | 0.12 | 0.729 |
|  | Female | 82 | 37 (45.1) | 45 (54.9) |  |  |
| Marital status | Single | 41 | 20 (48.8) | 21 (51.2) | 0.09 | 0.761 |
|  | Married | 182 | 84 (46.2) | 98 (53.8) |  |  |
| Ethnic group | Malay | 138 | 66 (47.8) | 72 (52.2) | 1.31 | 0.727 |
|  | Chinese | 52 | 22 (42.3) | 30 (57.7) |  |  |
|  | Indian | 18 | 10 (55.6) | 8 (44.4) |  |  |
|  | Others | 15 | 6 (40) | 9 (60) |  |  |
| Habits | None | 182 | 89 (48.9) | 93 (51.1) | 6.58 | 0.087 |
|  | Smoking | 34 | 15 (44.1) | 19 (55.9) |  |  |
|  | Alcohol | 2 | 0 (0) | 2 (100) |  |  |
|  | Smoking and Alcohol | 5 | 0 (0) | 5 (100) |  |  |
| Education level | None | 6 | 3 (50) | 3 (50) | 2.75 | 0.432 |
|  | Primary | 58 | 31 (53.4) | 27 (46.6) |  |  |
|  | Secondary | 133 | 56 (42.1) | 77 (57.9) |  |  |
|  | University | 26 | 14 (53.8) | 12 (46.2) |  |  |
| Employment | Unemployed | 138 | 76 (55.1) | 62 (44.9) | 10.35 | 0.001* |
|  | Employed | 85 | 28 (32.9) | 57 (67.1) |  |  |
| Diagnosis of COVID- 19 | No | 105 | 89 (84.8) | 16 (15.2) | 115.90 | 0.001* |
|  | Yes | 118 | 15 (12.7) | 103 (87.3) |  |  |
| COVID-19 category | Non-COVID | 105 | 89 (84.8) | 16 (15.2) | 116.74 | 0.001* |
|  | Category 1 | 8 | 2 (25) | 6 (75) |  |  |
|  | Category 2 | 16 | 2 (12.5) | 14 (87.5) |  |  |
|  | Category 3 | 26 | 2 (7.7) | 24 (92.3) |  |  |
|  | Category 4 | 57 | 8 (14) | 49 (86) |  |  |
|  | Category 5 | 11 | 1 (9.1) | 10 (90.9) |  |  |
| **Clinical characteristics** |  |  |  |  |  |  |
| Respiratory symptoms (fever, runny nose, sore throat, shortness of breath, cough) | No | 57 | 39 (68.4) | 18 (31.6) | 14.60 | 0.001* |
|  | Yes | 166 | 65 (39.2) | 101 (60.8) |  |  |
| Fever | No | 99 | 55 (55.6) | 44 (44.4) | 5.69 | 0.017* |
|  | Yes | 124 | 49 (39.5) | 75 (60.5) |  |  |
| Runny nose | No | 205 | 103 (50.2) | 102 (49.8) | 11.54 | 0.001* |
|  | Yes | 18 | 1 (5.6) | 17 (94.4) |  |  |
| Sore throat | No | 193 | 101 (52.3) | 92 (47.7) | 17.03 | 0.001* |
|  | Yes | 30 | 3 (10) | 27 (90) |  |  |
| Shortness of breath | No | 155 | 83 (53.5) | 72 (46.5) | 8.87 | 0.003* |
|  | Yes | 68 | 21 (30.9) | 47 (69.1) |  |  |
| Cough | No | 134 | 85 (63.4) | 49 (36.6) | 38.06 | 0.001* |
|  | Yes | 89 | 19 (21.3) | 70 (78.7) |  |  |
| Gastrointestinal symptoms (diarrhea, vomiting, abdominal pain, poor oral intake) | No | 207 | 91 (44) | 116 (56) | 6.87 | 0.009* |
|  | Yes | 16 | 13 (81.3) | 3 (18.8) |  |  |
| Diarrhea | No | 218 | 102 (46.8) | 116 (53.2) | 0.00 | 1.000 |
|  | Yes | 5 | 2 (40) | 3 (60) |  |  |
| Vomiting | No | 220 | 101 (45.9) | 119 (54.1) | 1.65 | 0.200 |
|  | Yes | 3 | 3 (100) | 0 (0) |  |  |
| Abdominal pain | No | 216 | 98 (45.4) | 118 (54.6) | 2.96 | 0.085 |
|  | Yes | 7 | 6 (85.7) | 1 (14.3) |  |  |
| Poor oral intake | No | 219 | 100 (45.7) | 119 (54.3) | 2.73 | 0.098 |
|  | Yes | 4 | 4 (100) | 0 (0) |  |  |
| Neurological symptoms (seizures, weakness, headache, dizziness) | No | 181 | 94 (51.9) | 87 (48.1) | 10.84 | 0.001* |
|  | Yes | 42 | 10 (23.8) | 32 (76.2) |  |  |
| Seizures | No | 220 | 102 (46.4) | 118 (53.6) | 0.01 | 0.906 |
|  | Yes | 3 | 2 (66.7) | 1 (33.3) |  |  |
| Weakness | No | 217 | 102 (47) | 115 (53) | 0.06 | 0.805 |
|  | Yes | 6 | 2 (33.3) | 4 (66.7) |  |  |
| Headache | No | 197 | 101 (51.3) | 96 (48.7) | 14.57 | 0.001* |
|  | Yes | 26 | 3 (11.5) | 23 (88.5) |  |  |
| Dizziness | No | 212 | 100 (47.2) | 112 (52.8) | 0.49 | 0.484 |
|  | Yes | 11 | 4 (36.4) | 7 (63.6) |  |  |
| Muscle and joint pain | No | 206 | 102 (49.5) | 104 (50.5) | 8.99 | 0.003* |
|  | Yes | 17 | 2 (11.8) | 15 (88.2) |  |  |
| **Laboratory investigations** |  |  |  |  |  |  |
| Hemoglobin g/dL | 12.0 - 15.0 | 99 | 43 (43.4) | 56 (56.6) | 0.73 | 0.392 |
|  | Abnormal | 124 | 61 (49.2) | 63 (50.8) |  |  |
| White cell count x 10^9^/L | 4.0 - 10.0 | 124 | 53 (42.7) | 71 (57.3) | 1.70 | 0.192 |
|  | Abnormal | 99 | 51 (51.5) | 48 (48.5) |  |  |
| Platelet x 10^9^/L | 150 - 410 | 167 | 78 (46.7) | 89 (53.3) | 0.00 | 0.971 |
|  | Abnormal | 56 | 26 (46.4) | 30 (53.6) |  |  |
| Sodium mmol/L | 136 - 145 | 115 | 61 (53) | 54 (47) | 3.92 | 0.048* |
|  | Abnormal | 108 | 43 (39.8) | 65 (60.2) |  |  |
| Potassium mmol/L | 3.5 - 5.1 | 176 | 87 (49.4) | 89 (50.6) | 2.62 | 0.105 |
|  | Abnormal | 47 | 17 (36.2) | 30 (63.8) |  |  |
| Urea mmol/L | 2.5 - 6.7 | 129 | 59 (45.7) | 70 (54.3) | 0.10 | 0.752 |
|  | Abnormal | 94 | 45 (47.9) | 49 (52.1) |  |  |
| Creatinine µmol/L | 50.4 - 98.1 | 124 | 53 (42.7) | 71 (57.3) | 1.70 | 0.192 |
|  | Abnormal | 99 | 51 (51.5) | 48 (48.5) |  |  |
| Total protein g/L | 64 - 83 | 170 | 75 (44.1) | 95 (55.9) | 1.82 | 0.177 |
|  | Abnormal | 53 | 29 (54.7) | 24 (45.3) |  |  |
| Albumin g/L | 34 - 48 | 106 | 47 (44.3) | 59 (55.7) | 0.43 | 0.513 |
|  | Abnormal | 117 | 57 (48.7) | 60 (51.3) |  |  |
| Bilirubin µmol/L | 3.4 - 20.5 | 183 | 88 (48.1) | 95 (51.9) | 0.86 | 0.353 |
|  | Abnormal | 40 | 16 (40) | 24 (60) |  |  |
| Alanine transaminase IU/L | 0 - 55 | 180 | 88 (48.9) | 92 (51.1) | 1.90 | 0.168 |
|  | Abnormal | 43 | 16 (37.2) | 27 (62.8) |  |  |
| Alkaline phosphatase IU/L | 40 - 150 | 191 | 90 (47.1) | 101 (52.9) | 0.13 | 0.724 |
|  | Abnormal | 32 | 14 (43.8) | 18 (56.3) |  |  |

*Significant P<0.05 χ^2^ Chi-square test

Table 4 Univariate and multivariate logistic regression analysis for anxiety

| Variables |  | Univariate |  |  | Multivariate |  |
| --- | --- | --- | --- | --- | --- | --- |
|  | OR | 95% CI for EXP (B) | p | OR | 95% CI  for EXP (B) | p |
| Diabetes mellitus | 0.51 | 0.29- 0.88 | 0.015 * | 0.71 | 0.30-1.68 | 0.434 |
| Employment | 2.50 | 1.42- 4.38 | 0.001 * | 1.86 | 0.75-4.61 | 0.178 |
| COVID-19 diagnosis | 38.20 | 17.87-81.62 | 0.001 * | 36.92 | 17.09-79.78 | 0.001* |
| Respiratory symptoms | 3.37 | 1.78 - 6.38 | 0.001 * | 1.74 | 0.34-8.77 | 0.504 |
| Fever | 1.91 | 1.12-3.27 | 0.018 * | 0.40 | 0.12-1.37 | 0.144 |
| Cough | 6.39 | 3.45-11.84 | 0.001 * | 1.74 | 0.63-4.77 | 0.284 |
| Sore throat | 9.88 | 2.90-33.66 | 0.001 * | 0.59 | 0.11-3.04 | 0.526 |
| Shortness of breath | 2.58 | 1.41-4.72 | 0.002 * | 0.54 | 0.18-1.63 | 0.275 |
| Runny nose | 17.17 | 2.24-131.40 | 0.006 * | 2.193 | 0.22-22.20 | 0.506 |
| Neurological symptoms | 3.46 | 1.60-7.45 | 0.002 * | 2.94 | 1.03-8.41 | 0.044 * |
| Headache | 8.07 | 2.35-27.74 | 0.001 * | 0.52 | 0.06-4.87 | 0.566 |
| Gastrointestinal symptoms | 0.18 | 0.05- 0.65 | 0.009 * | 0.24 | 0.04-1.56 | 0.137 |
| Muscle and joint pain | 7.36 | 1.64-32.98 | 0.009 * | 1.40 | 0.17-11.16 | 0.753 |
| Sodium | 0.59 | 0.34-1.00 | 0.049 * | 1.66 | 0.72-3.84 | 0.239 |

*Significant P<0.05 OR Odds ratio, 95% CI, 95% confidence interval
